# Sleep duration and the risk of prostate cancer: the Ohsaki Cohort Study

**DOI:** 10.1038/sj.bjc.6604425

**Published:** 2008-06-10

**Authors:** M Kakizaki, K Inoue, S Kuriyama, T Sone, K Matsuda-Ohmori, N Nakaya, S Fukudo, I Tsuji

**Affiliations:** 1 Division of Epidemiology, Department of Public Health and Forensic Medicine, Tohoku University Graduate School of Medicine, Sendai, Japan; 2 Division of Behavioral Medicine, Department of Functional Medical Science, Tohoku University Graduate School of Medicine, Sendai, Japan

**Keywords:** sleep duration, prostate cancer, incidence, Japanese, prospective cohort study

## Abstract

In a prospective study of prostate cancer incidence (127 cases), among 22 320 Japanese men, sleep duration was associated with lower risk; the multivariate hazard ratio of men who slept ⩾9 h per day compared with those who slept less was 0.48 (95% confidence interval: 0.29–0.79, *P* for trend=0.02).

Sleep duration is associated with various health outcomes (Youngstedt and Kripke, 2004) including three observational studies of sleep duration and breast cancer (Verkasalo *et al*, 2005; McElroy *et al*, 2006; Pinheiro *et al*, 2006). Melatonin, which is secreted mainly from the pineal gland and plays a role in sleep duration, is suggested as one of the candidates responsible for the association in breast cancer (Brzezinski, 1997; Schernhammer and Schulmeister, 2004) as it influences the synthesis and secretion of sex hormones by promoting the release of gonadotropin-releasing hormone (Martin and Klein, 1976; Aleandri *et al*, 1996).

In relation to melatonin, there have been several observational studies of night work, shift work or visual impairment, and sex hormone-related cancers such as prostate or breast (Feychting *et al*, 1998; Verkasalo *et al*, 1999; Kliukiene *et al*, 2001; Megdal *et al*, 2005; Kubo *et al*, 2006; Conlon *et al*, 2007; Schwartzbaum *et al*, 2007). However, there has been no study of sleep duration and prostate cancer risk, even though prostate cancer, like breast cancer, is also a sex hormone-related cancer.

We therefore examined the association between sleep duration and prostate cancer risk in a population of Japanese men, in whom the mortality of prostate cancer is increasing (Statistics and Information Department, Minister's Secretariat, Ministry of Health Labour and Welfare of Japan, 2007).

## Materials and methods

Details of the Ohsaki National Health Insurance (NHI) Cohort Study have been described previously (Tsuji *et al*, 1998; Kuriyama *et al*, 2006). Briefly, this prospective cohort study was started in 1994 and included 26 481 men aged 40–79 years living in the 14 municipalities of Miyagi Prefecture, northeastern Japan. The response rate was 94.0% (*N*=24 895) for the questionnaire that was self-administered and included items about sleep duration and other health-related lifestyle factors. The study protocol was reviewed and approved by the ethics committee of Tohoku University School of Medicine.

After exclusion of subjects who had withdrawn from the NHI before follow-up, those who had a history of cancer, those who had omitted responses for sleep duration, and those who had reported sleep duration of less than 4 h or more than 12 h, 22 320 subjects remained. To follow-up participants for mortality and migration, we reviewed the NHI withdrawal history files for 1995–2001. Through the Miyagi Prefectural Cancer Registry, we identified 127 incident cases of prostate cancer. During the study period, there had been no mass screening programme for prostate cancer in this area. Clinical stage was classified according to the TNM system as localised (T1–T2), advanced (T3–T4), metastatic (N+ and/or M+), or unknown.

With regard to the sleep duration, participants answered the mean integer number of hours of sleep per day during the last year. Because of the small number who slept for less than 7 h and more than 8 h, we categorised sleep duration into three groups: ⩽6, 7–8, and ⩾9 h per day. We estimated hazard ratios (HRs) and 95% confidence intervals (CIs) of prostate cancer incidence according to sleep duration, using the Cox proportional hazards model, with adjustment for age and potential confounders. The continuous *P* for trend was calculated by treating sleep duration as a continuous variable, and the categorical *P* for trend by treating each category as a continuous variable. Interactions between the risk and all confounders were tested through the addition of cross-product terms to multivariate model.

All statistical analyses were performed using SAS statistical software, version 9.1 (SAS Institute Inc, Cary, NC, USA), and all those reported were two-sided; differences at *P*-values of <0.05 were accepted as significant.

## Results

Table 1 shows the baseline characteristics of subjects according to sleep duration. Subjects who slept 9 h or more per day (long sleepers) were older, less likely to be employed and married, and more likely to have a history of disease. Those who slept 6 h or less per day (short sleepers) were more likely to have never smoked and less likely to walk more than 1 h per day.

We found an inverse association between sleep duration and risk. The HR of prostate cancer in short sleepers was 1.34 (95% CI: 0.83–2.17) and the HR for long sleepers was 0.48 (95% CI: 0.29–0.79) (*P* for trend=0.02). This result did not change substantially when subjects whose event occurred within 3 years of baseline (*N*=46) were excluded and analysis of each clinical stage (localised or advanced/metastatic) was examined (Table 2).

In addition, we examined in detail, confounding and effect modification by age and other covariates on the associations with sleep duration. No significant interaction was observed between sleep duration and other confounding factors for risk on a multiplicative scale (data not shown).

## Discussion

In the first study to address the question, we found an inverse association between sleep duration and the risk of prostate cancer in Japanese men. There have, however, been three observational studies of sleep duration and breast cancer risk (McElroy *et al*, 2006; Pinheiro *et al*, 2006). Among these, one reported a decreased risk in long sleepers (Verkasalo *et al*, 2005), the second reported no association (Pinheiro *et al*, 2006), whereas the third reported an increased risk in long sleepers (McElroy *et al*, 2006). Among possible reasons for these differences from our findings (apart from the site of cancer), McElroy *et al* (2006) conducted a case–control study while Pinheiro *et al* (2006) studied residential nurses in the United States with rotating shift work and varying timing of sleep so that generalising from their results may be inappropriate.

Melatonin may be a factor in these inverse associations with sex hormone-related cancers (Brzezinski, 1997; Schernhammer and Schulmeister, 2004; Shiu, 2007). A decreased sleep duration results in a shorter duration of nocturnal melatonin secretion (Wehr, 1991). Melatonin may have an inhibitory effect on gonadal function, including the synthesis and secretion of sex hormones, by promoting the release of gonadotropin-releasing hormone (Martin and Klein, 1976; Aleandri *et al*, 1996). It also exerts an antiproliferative effect on prostate and breast cancer cell lines (Shiu, 2007).

Strengths of the present study include its prospective nature and its base in the general population. In addition, the Miyagi Prefectural Cancer Registry is one of the earliest and most accurate population-based cancer registries in Japan (Takano and Okuno, 1997); so our data on cancer incidence are considered sufficiently reliable.

Methodological limitations include the fact that sleep duration was self-reported. Second, we had no information about sleep quality, the timing of sleep, the use of sleep medication, the presence of sleeping disorders or rotating shift work. Such factors influence sleep duration and thereby might affect the findings.

In conclusion, we have found a significant inverse association between sleep duration and the risk of prostate cancer incidence in Japanese men.

## Figures and Tables

**Table 1:** Baseline characteristics of the subjects according to sleep duration

**Sleep duration**	**⩽6**	**7–8**	**⩾9**
Number of subjects	2671	15 127	4522
Mean age, s.d. (years)	57.7 (11.0)	58.0 (10.4)	64.0 (9.3)
Mean body mass index, s.d. (kg m^−2^)	23.7 (3.2)	23.4 (2.9)	23.1 (3.4)
Having history of diseases^a^ (%)	31.3	29.4	38.9
Employed (%)	59.2	61.0	47.8
Married (%)	80.1	82.2	77.0
Never smoked (%)	19.4	18.2	15.9
Never drank (%)	15.5	16.3	14.8
Walking 1 h per day or more (%)	42.6	45.7	43.4
Having family history of cancer (%)	30.0	29.3	28.4

s.d.=standard deviation.

a History of stroke, hypertension, myocardial infarction, or diabetes mellitus.

**Table 2:** Cox proportional hazard ratios (HRs) for prostate cancer incidence by sleep duration in Japanese men

	**Sleep duration (hours/ per day)**				
	**⩽6**	**7–8**	**⩾9**	***P* for trend^a^**	***P* for trend^b^**
Person-years	16 716	94 786	27 104		
*All prostate cancer*					
Number of cases	21	87	19		
Age-adjusted HR (95% CI)	1.34 (0.83–2.15)	1.00 (reference)	0.46 (0.28–0.75)	0.01	0.0003
Multivariate HR1 (95% CI)^c^	1.34 (0.83–2.17)	1.00 (reference)	0.48 (0.29–0.79)	0.02	0.0007
Multivariate HR2 (95% CI)^d^	1.38 (0.77–2.48)	1.00 (reference)	0.36 (0.18–0.72)	0.01	0.0009
*Localised prostate cancer*					
Number of cases	4	19	3		
Multivariate HR1 (95% CI)^c^	1.13 (0.38–3.35)	1.00 (reference)	0.29 (0.09–0.997)	0.11	0.05
*Advanced or metastatic prostate cancer*					
Number of cases	8	25	8		
Multivariate HR1 (95% CI)^c^	1.82 (0.82–4.05)	1.00 (reference)	0.79 (0.35–1.77)	0.30	0.12

CI=confidence interval.

a *P* for trend values were calculated by treating sleep duration as a continuous variable.

b *P* for trend values were calculated by treating each category of sleep duration as a continuous variable.

c Multivariate HR1 was adjusted for age (<45, 45–49, 50–54, 55–59, 60–64, 65–69, 70–74, and 75+ years old); marital status (married or unmarried); education (junior high school or less, high school, college/university or higher); job status (employed or unemployed); history of diseases (history of stroke, hypertension, myocardial infarction, or diabetes mellitus); family history of cancer (presence or absence in first-degree relatives); body mass index (<18.5, 18.5–24.9, or ⩾25.0 kg m^−2^); cigarette smoking (never smoked, smoked in the past, currently smoking 1–19 cigarettes per day, or currently smoking ⩾20 cigarettes per day); alcohol consumption (never drank alcohol, drank in the past, or currently drinking); walking status (<1 h per day or ⩾1 h per day).

d Multivariate HR2 was estimated excluding 46 subjects who were diagnosed with prostate cancer within the first 3 years from baseline and was adjusted for using the same variables as in multivariable HR1.
